# Delivery of Cinnamic Aldehyde Antioxidant Response Activating nanoParticles (ARAPas) for Vascular Applications

**DOI:** 10.3390/antiox10050709

**Published:** 2021-04-29

**Authors:** Ana E. Cartaya, Halle Lutz, Sophie Maiocchi, Morgan Nalesnik, Edward M. Bahnson

**Affiliations:** 1 Department of Surgery, Division of Vascular Surgery, University of North Carolina at Chapel Hill, Chapel Hill, NC 27599, USA; acartaya@email.unc.edu (A.E.C.); sophie_maiocchi@med.unc.edu (S.M.); 2 Center for Nanotechnology in Drug Delivery, University of North Carolina at Chapel Hill, Chapel Hill, NC 27599, USA; hjlutz@ncsu.edu; 3 Department of Pharmacology, University of North Carolina at Chapel Hill, Chapel Hill, NC 27599, USA; 4 McAllister Heart Institute, University of North Carolina at Chapel Hill, Chapel Hill, NC 27599, USA; 5 Curriculum in Toxicology & Environmental Medicine, University of North Carolina at Chapel Hill, Chapel Hill, NC 27599, USA; nalesnik@live.unc.edu; 6 Department of Cell Biology & Physiology, University of North Carolina at Chapel Hill, Chapel Hill, NC 27599, USA

**Keywords:** vascular nanomedicine, nanotherapeutics, nanoantioxidants, reactive oxygen species, Nfr2 activators, pluronic micelles

## Abstract

Selective delivery of nuclear factor erythroid 2-related factor 2 (Nrf2) activators to the injured vasculature at the time of vascular surgical intervention has the potential to attenuate oxidative stress and decrease vascular smooth muscle cell (VSMC) hyperproliferation and migration towards the inner vessel wall. To this end, we developed a nanoformulation of cinnamic aldehyde (CA), termed Antioxidant Response Activating nanoParticles (ARAPas), that can be readily loaded into macrophages ex vivo. The CA-ARAPas-macrophage system was used to study the effects of CA on VSMC in culture. CA was encapsulated into a pluronic micelle that was readily loaded into both murine and human macrophages. CA-ARAPas inhibits VSMC proliferation and migration, and activates Nrf2. Macrophage-mediated transfer of CA-ARAPas to VSMC is evident after 12 h, and Nrf2 activation is apparent after 24 h. This is the first report, to the best of our knowledge, of CA encapsulation in pluronic micelles for macrophage-mediated delivery studies. The results of this study highlight the feasibility of CA encapsulation and subsequent macrophage uptake for delivery of cargo into other pertinent cells, such as VSMC.

## 1. Introduction

Cardiovascular disease remains the leading cause of death and disability worldwide, placing a heavy burden on human and economic welfare [1]. Atherosclerosis, or plaque buildup in arteries, is the primary underlying condition driving cardiovascular disease [1]. Surgical procedures to restore luminal diameter and blood flow in severely affected vessels include balloon angioplasty with or without stent placement [2]. Incidence rates of restenosis in patients undergoing coronary stenting are approximately 30% for bare metal stenting and 15–12% for latest generation drug-eluting stents (DES) at 12 months, in contrast with up to 50% restenosis rates in patients undergoing balloon angioplasty without stenting [2,3]. Restenosis is a complex clinical complication that occurs as a consequence of neointimal hyperplasia, characterized by the proliferation and migration of vascular smooth muscle cells (VSMC) and fibroblasts towards the inner vessel wall [3,4]. For candidate vessels, DES are the most effective treatment [3]. DES are loaded with anti-proliferative drugs, which exert their effect indiscriminately in the area of stent placement, leading to delayed arterial healing, and increased incidence of neoatherosclerosis formation [2]. As such, therapies aimed to selectively inhibit VSMC proliferation and migration after surgical intervention are highly sought after.

The pathological proliferation and migration of VSMC in neointimal hyperplasia have been associated with local disturbance of vessel redox homeostasis after surgery [5,6]. Specifically, the overproduction of reactive oxygen species (ROS) like superoxide, produced via NAD(P)H oxidase 1 (NOX1) activity, and hydrogen peroxide, typically produced from superoxide dismutation, activates proliferative and migratory signals in VSMC [5,6]. Hence, small molecules that aim to restore redox homeostasis in VSMC are attractive therapeutics for the prevention of restenosis. Many antioxidant interventions have focused on scavenging reactive oxygen species indiscriminately. Targeting the nuclear factor erythroid 2-related factor 2 (Nrf2) allows the cell to express an array of different antioxidant defenses and deliver them to specific subcellular compartments. This is an innovative approach compared to small-molecule scavenger antioxidant therapies. Hence, Nrf2 activators are an active area of research. Nrf2 is a transcription factor and known master regulator of the cellular antioxidant response [7]. Nrf2 activation results in a rapid increase and accumulation of antioxidant enzymes such as glutathione peroxidase, which neutralize ROS responsible for VSMC activation [7]. Studies have shown that the use of Nrf2 activators is a feasible approach to inhibit restenosis in animal models [8,9,10]. Cinnamic aldehyde (CA) is the main constituent of cinnamon oil, extracted from cinnamon tree bark [11]. CA is an α,β-unsaturated carbonyl compound; hence, an electrophile that reacts with thiols to form stable protein adducts [11]. CA reacts with Cys151 in Keap 1 thus, belonging to the Class I of Nrf2 activators [12]. CA exhibits a range of vasculoprotective effects, including: inhibition of VSMC proliferation and migration [9,13], increased bioavailability on endothelial nitric oxide, and increased vasodilation [14,15], reduction of vascular inflammation [13,16,17], and atherosclerosis burden [17]. Our group recently showed that CA inhibits restenosis in a diabetic rat model when applied locally to the vessel [9]. However, a major limitation of antioxidant therapies is that systemic delivery has shown only modest results in clinical studies [18]. This highlights the need for a system targeted to the area of surgical injury in the vessel in which to deliver antioxidant interventions.

Nanotechnology has revolutionized the field of drug delivery for therapeutic applications. Active targeting strategies for nanotherapeutics focus on immunotargeting to inflammatory markers such as cell adhesion molecules, or to exposed extracellular matrix components [19]. Targeting strategies employing autologous drug-loaded immune cells have been successful in treating neuroinflammation in mice models [20,21] but have never been explored in the context of vascular disease. Indeed, briefly after vascular intervention, macrophages are recruited to the injury site where they infiltrate the vessel [2], making them an attractive delivery strategy. The phagocytic capacity of macrophages allows them to engulf a multitude of particles, including antioxidant encapsulated nanoparticles (NP) [20,21]. This approach, termed “Trojan Horse,” has the advantages of cargo integrity protection from degradation inside and outside host cells [20,21]. Targeted delivery has been the subject of much research in the cancer field (>14,000 Pubmed articles between 2000–2021). However, not much has been done in cardiovascular disease research (<2000 Pubmed articles in the same period). Coupling Nrf2 activation with cell-mediated nanoparticle delivery is a highly innovative approach to overcome the issue of systemic redox interventions. In particular, CA would benefit from this drug encapsulation approach given its poor solubility in water, and high reactivity [22]. Thus, in this study, we aimed to create CA-loaded nanoparticles, termed Antioxidant Response Activating nanoParticles (ARAPas), and study their potential for macrophage-mediated prevention of pathological VSMC proliferation and migration. We hypothesized that CA will interact with pluronic polymers (also known under their non-proprietary name poloxamers), to form drug-loaded micelles that once taken up by macrophages ex vivo will deliver antioxidant cargo to VSMC to activate Nrf2 and inhibit ROS-mediated proliferation and migration in vitro.

## 2. Materials and Methods

### 2.1. Materials

Synperonic^®^ PE/Pluronic P84 (P84,713538), Pluronic^®^ F-127 (F127, P2443), (Sigma-Aldrich, St. Louis, MO, USA), Poloxamer 331/Pluronic^®^ L101 (P1608) (Spectrum Chemical, Gardena, CA, USA). Nile red (N0659, TCI America, Portland, OR, USA), cinnamic aldehyde (CA) (C80687; Sigma-Aldrich, St. Louis, MO, USA), Rhodamine 123 (83702, Sigma-Aldrich, St. Louis, MO, USA), DiD (1,1′-Dioctadecyl-3,3,3′,3′-Tetramethylindodicarbocyanine Perchlorate) (D307, Thermo-Fisher, Waltham, MA, USA), (MTT) 3-(4,5-Dimethyl-2-thiazolyl)-2,5-diphenyl-2H-tetrazolium bromide (L11939, Alfa Aesar, Haverhill, MA, USA).

### 2.2. Cell Culture

RAW 264.7 (TIB-71, ATCC, Manassas, Virginia, USA) were cultured in high glucose DMEM (11995065, Gibco, Gaithersburg, MA, USA) supplemented with 10% heat-inactivated fetal bovine serum (FBS) (10438034, Gibco, Gaithersburg, MA, USA) and 1X penicillin-streptomycin (30-002-CI, Corning, Corning, NY, USA). THP-1 (TIB-202, ATCC, Manassas, VA, USA) were cultured in RPMI 1640 (11875168, Gibco, Gaithersburg, MA, USA) supplemented with 10% FBS (Gibco, Gaithersburg, MA, USA) and 1X penicillin-streptomycin (30-002-CI, Corning, Corning, NY, USA). Additionally, THP-1 cells were supplemented with 162 nM of phorbol myristate acetate (PMA) (524400, Sigma-Aldrich, St. Louis, MO, USA) for 48 h to stimulate terminal differentiation. Primary rat aortic smooth muscle cells (RASMC) from Sprague Dawley rats (10–12 weeks old), were isolated from thoracic aortae as previously described using an explant method [23]. RASMC were used between passages 4–9 for all experiments. RASMC were cultured in low glucose DMEM (11885092, Gibco, Gaithersburg, MA, USA) and Ham’s F-12 Nutrient Mix (11765054, Gibco, Gaithersburg, MA, USA) in a 1:1 ratio, supplemented with 10% heat-inactivated FBS (10438034, Gibco, Gaithersburg, MA, USA), 5 mM glutamine (25030081, Gibco, Gaithersburg, MA, USA), and 1X penicillin-streptomycin (30-002-CI, Corning, Corning, NY, USA).

### 2.3. Critical Micellar Concentration with Nile Red Assay

Critical Micellar Concentration (CMC) using nile red probe was determined as previously described [24]. Briefly, nile red was dissolved in solvent to 300 nM with or without the addition of micellar triblock copolymers at concentrations ranging from 0–10% (*w/v*). Using a microplate spectrophotometer (Biotek, Winooski, VT, USA), nile red fluorescence was measured between 575 and 700 nm at room temperature in phosphate buffer saline pH 7.4. The shift that occurs in maximum emission wavelength peak between nile red in solvent versus nile red in micelles was calculated and represented as change in maximum emission at different concentrations of micellar triblock copolymer. For the data that formed two distinct lines, the CMC was calculated as the intersect of two linear fits. For the data in which transition was too steep to provide distinct lines, a sigmoidal fit was applied, and the second derivative was used to calculate the CMC.

### 2.4. Cinnamic Aldehyde Loaded ARAPas Synthesis

Cinnamic aldehyde- (CA-) was loaded into pluronic micelles (PM)using a one-step assembly method based on direct dissolution. CA was initially dissolved in DMSO to a concentrated stock. CA was then further diluted in 20 nm-filtered PBS or water to the desired final concentration prior to addition of the pluronic solution. Pluronic L101 was dissolved in 20 nm filtered distilled water to 15% (*w/v*) and the stock added to the CA solution at the appropriate volume to reach the desired final percent (*w/v*). The solution was vortexed for one minute, and then left undisturbed for at least an hour to allow for sample equilibration.

### 2.5. Nanoparticle Size and Shape Determination

Average particle size, and polydispersity index (PdI) were determined using dynamic light scattering (DLS) and confirmed using nanotracking analysis (NTA). Samples of CA-ARAPas or PM alone were diluted in 20 nm filtered PBS to 0.0005% (DLS) or 0.001% (NTA) pluronic L101. DLS samples were analyzed at room temperature using a Malvern Zetasizer (Nano-ZS; Malvern Instruments, Malvern, UK) using 173° backscatter angle. NTA samples were analyzed using a NanoSight NS500 (Malvern Panalytical, Malvern, UK).

Conventional TEM images were taken on an FEI Tecnai T-12 TEM (ThermoFisher Scientific, Waltham, MA, USA) at 80 kV with an Orius 2k × 2k CCD camera (Gatan, Inc., Pleasanton, CA, USA). CA-ARAPas at 0.01% PL101 and 100 µM CA in PBS were prepared for TEM by negative staining. Briefly, 8 μL samples were incubated onto 400 mesh copper grids covered with a thin carbon film. After 3 min, samples were stained with 2% uranyl acetate for 3–5 min and air-dried before imaging.

### 2.6. Surface Charge Determination

Surface charge or zeta potential of the particles was determined using a Malvern Zetasizer (Nano-ZS; Malvern Instruments, Malvern, UK). Samples were prepared at 0.05% *w/v* of pluronic L101 in 20 nm filtered distilled water.

### 2.7. Determination of Drug Entrapment Efficiency

Entrapment of cinnamic aldehyde in ARAPas was determined by measuring the amount of drug in the supernatant after centrifugation at 10,000 rpm for 30 min and comparing to a standard cinnamic aldehyde curve. Absorbance was measured at 286 nm with a microplate spectrophotometer (Epoch, Biotek, Winooski, VT, USA), and encapsulation entrapment (EE) determined using Equation (1):

(1) $$\mathrm{EE}=\;\frac{weight\;of\;CA\;in\;the\;micelle}{theoretial\;weigh\;of\;encapsulated\;CA}\times 100$$

### 2.8. Wound Healing Assay

Relative migration percent was determined using a standard scratch wound healing assay. RASMC were seeded at 50,000 cells per well in a 12-well plate. Cells were then synchronized in serum-free media for 24 h before treatment. Cells in the scratch area were imaged using a cell imaging multi-mode reader (Cytation 5, Biotek, Winooski, VT, USA) at the start of ccc were seeded at 5000 cells per well in 96-well plates. Cells were then synchronized in serum-free media for 24 h before treatment. RASMC were treated with pluronic L101 concentrations 0–1% (*w/v*), CA and CA-ARAPas at concentrations 0–600 µM of CA with a standard 0.01% (*w/v*) of pluronic L101 for micelles. Cells were exposed to the listed treatments for 24 h, after which they were switched to a 0.4 mg/mL MTT solution in media and incubated for 4 h. MTT solution was discarded, and the plates were left to air-dry overnight. Crystals formed were dissolved in DMSO, and the absorbance was measured at 560 nm with a background of 670 nm using a microplate spectrophotometer (Epoch, Biotek, Winooski, VT, USA).

### 2.9. Western Blot

Synchronized RASMC (seeded at 1.5 × 10^6^ cells in 100 mm, TC treated plates) were treated for 4 h with 0.05% pluronic L101, 100 µM CA, 100 µM CA-loaded pluronic L101 (0.05%) micelle. Whole-cell lysates were collected mechanically using a rubber policeman. Protein was quantified by the BCA assay (23225; Thermo-Fisher Scientific, Waltham, MA, USA). Protein (10 µg) was loaded in a 4% stacking/13% running homecast gel, following Bio-Rad protocol. Proteins were transferred to a PVDF membrane (10600023; GE Life Sciences, Marlborough, MA, USA). The membrane was submerged overnight in Near Infrared Blocking Buffer (RLMB-070; Rockland Immunochemicals, Pottstown, PA, USA), which was also used for all protein probing. Primary antibodies were used at dilutions of 1:1000 of anti-Nrf2 antibody (ab137550; Abcam, Cambridge, UK), and 1:15,000 of anti-β-actin antibody (A5441; Sigma-Aldrich, St. Louis, MO, USA). Secondary antibodies used at dilutions of 1:5000 for anti-rabbit (926-32211; LI-COR Biosciences, Lincoln, NE, USA) and 1:10,000 for anti-mouse (926-68070; LI-COR Biosciences, Lincoln, NE, USA). Membranes were imaged using LI-COR Odyssey 9120 Infrared Imaging System (LI-COR Biosciences, Lincoln, NE, USA).

### 2.10. Superoxide Dismutase Activity Assay

Synchronized RASMC (seeded at 2 × 105 cells per well in 6 well plates) were treated for 24 h with complete media alone or supplemented with 0.01% pluronic L101, 100 µM CA, 100 µM CA- loaded pluronic L101 (0.01%) micelle. Whole-cell lysates from 2 wells per condition were collected mechanically and lysed via sonication without the use of protease inhibitors. Superoxide dismutase (SOD) activity assay was performed according to the manufacturer’s instructions (706002, Cayman Chemical, Ann Arbor, MI, USA) in a UV 96-well plate. Absorbance was measured using a cell imaging multi-mode reader (Cytation 5, Biotek, Winooski, VT, USA).

### 2.11. Total Glutathione Assay

Synchronized RASMC (seeded at 2 × 105 cells per well in 6 well plates) were treated for 24 h with complete media alone or supplemented with 0.01% pluronic L101, 100 µM CA, 100 µM CA- loaded pluronic L101 (0.01%) micelle. Whole-cell lysates from 2 wells per condition were collected mechanically and lysed via sonication without the use of protease inhibitors. Total glutathione (GSH) was quantified according to the manufacturer’s instructions (703002, Cayman Chemical, Ann Arbor, MI, USA) in a UV 96-well plate using the endpoint method. Absorbance was measured using a cell imaging multi-mode reader (Cytation 5, Biotek, Winooski, VT, USA).

### 2.12. Macrophage Uptake of Fluorescent Micelles Kinetic

To obtain fluorescent micelles, Rhodamine-123 was dissolved in phenol-free macrophage media to 5 µM, and pluronic L101 added to a final concentration of 0.01% (*w*/*v*). RAW 264.7 and THP-1 cells (seeded at 1 × 104 cell in 96-well imaging plates, 655891, Greiner Bio-One, Monroe, NC, USA) were treated with Rhodamine-loaded micelles, or with Rhodamine alone for up to 2 h. After treatment, cells were stained with Hoescht 33,342 10 µg/mL and incubated for 10 min. A cell imaging multi-mode reader (Cytation 5, Biotek, Winooski, VT, USA) was used to image the entire wells in both green (Rhodamine) and blue (Hoescht) channels. Gen5 software was used to determine the number of cells containing Rhodamine compared to the total amount of cells as determined by the nuclear stain and represented as percent of cells positive for Rhodamin-123.

### 2.13. Macrophage Uptake of Fluorescent Micelles Confocal Microscopy

To obtain fluorescent micelles, DiD was dissolved in macrophage media to 5 µM and pluronic L101 added to a final concentration of 0.01% (*w*/*v*). RAW 264.7 and THP-1 cells (seeded at 2 × 104 cell per well in a Nunc™ Lab-Tek™ II 8 well chamber slide system, 12-565-8, Thermo Scientific, Waltham, MA, USA) were treated with DiD-loaded micelles, or with DiD alone for 30 min. The cell membrane was stained using MemBrite^®^ Fix Staining (30095, Biotium, Fremont, CA, USA) following the manufacturer’s instructions. Cells were fixed in 2% paraformaldehyde, and permeabilized with 0.1% Triton-X (X100; Sigma-Aldrich, St. Louis, MO, USA). Cells were then stained with 0.0012 µM DAPI (D3571; Invitrogen, Carlsbad, CA, USA) and mounted with ProLong™ Gold Antifade Mountant (P36930, Thermo Scientific, Waltham, MA, USA). Three-dimensional image acquisition was done using a LSM 780 laser scanning confocal microscope (Carl Zeiss Microscopy, Jena, Germany). Three-dimensional reconstructions were carried out using Imaris v9.5.0 (Bitplane AG, Zürich, Switzerland).

### 2.14. Measurement of Nitrite in Complete Media Samples

Murine RAW 264.7 macrophages (seeded at 2 × 104 cells in 96-well glass bottom imaging plate, 655891, Greiner Bio-One, Monroe, NC, USA) were allowed to attach overnight in high glucose DMEM (11995065, Gibco, Gaithersburg, MA, USA) supplemented with 10% heat-inactivated fetal bovine serum (FBS) (10438034, Gibco, Gaithersburg, MA, USA) and 1x penicillin-streptomycin (30-002-CI, Corning, Corning, NY, USA). Cells were primed with IFN-γ (10 ng/mL, 485-MI, R&D Systems, Minneapolis, MN, USA) for 7 h, prior to addition of treatments (vehicle, cinnamic aldehyde or cinnamic aldehyde micelles (50–100 μM)) with LPS (100 ng/mL, L4391-1MG, Sigma-Aldrich, St. Louis, MO, USA) as previously described [25]. Cells were treated for 24 h, at which point the media was removed for analysis of nitrite levels. Cells were washed with ice-cold HBSS, and then fixed with 2% PFA in PBS for 15 min. Cells were stained with 0.0012 µM DAPI (D3571; Invitrogen, Carlsbad, CA, USA) for 5 min and using a cell imaging multi-mode reader (Cytation 5) to obtain the total cell count per well. Nitrite in complete media samples was analyzed with the Sievers 280i Nitric Oxide Analyzer (NOA) using the tri-iodide reduction, and the corresponding packaged software Liquid.vi as per the ^●^NO Analyzer manufacturer instructions. Acidic iodide reducing reagent was prepared fresh (50 mg potassium iodide, 2 mL distilled H_2_O, and 5 mL glacial acetic acid). A standard curve was constructed with sodium nitrite solutions prior to sample measurements. Complete media samples were diluted with ice-cold distilled water (1 volume of complete media to 1 volume of distilled water). An amount 50 μL of diluted complete media samples were injected into the reducing reagent, and the area under the peak was used to measure the content of nitrite in picomoles.

### 2.15. Fluorescent Micelle Loaded Macrophage-RASMC Co-Culture

Synchronized RASMC (seeded at 5 × 103 cells in 96 well glass bottom imaging plate, 655891, Greiner Bio-One, Monroe, NC, USA) were treated with DiD micelle-loaded and empty RAW 264.7 macrophages at a ratio of 1:10 RASMC to macrophages. Macrophages were pretreated with 5 µM DiD or DiD-ARAPas for 2 h. Control RASMC were treated with 250 nM of DiD, which is equivalent to the amount of DiD loaded in 5 × 10^4^ macrophages. Cells were co-cultured in phenol-free media 1:1 high glucose DMEM:F12 (21041025, Gibco, Gaithersburg, MA, USA). Cells were imaged every hour for 24 h using a cell imaging multi-mode reader (Cytation 5) equipped with a CO_2_ chamber. For confocal microscopy, synchronized RASMC (seeded at 2 × 10^4^ cells in 96-well glass bottom imaging plate, 655891, Greiner Bio-One, Monroe, NC, USA) were treated with DiD-labeled micelle-loaded and also empty RAW 264.7 macrophages at a ratio of 1:10 RASMC to macrophages. Macrophages were pretreated with 5 µM DiD-ARAPas for 2 h. RASMC were also treated with 1 µM of DiD, equivalent to the amount of DiD loaded in 2 × 10^5^ macrophages at the same volume. After 24 h, cells were fixed in 2% paraformaldehyde, and permeabilized with 0.1% Triton-X (X100; Sigma-Aldrich). Cells were then probed for alpha smooth muscle actin (1:200, 48938; Cell Signaling Technology, Danvers, MA, USA) followed by AlexaFluor555 goat anti-mouse IgG (2 µg/mL, A21425; Thermo-Fisher Scientific, Waltham, MA, USA). All antibodies were diluted in IHC-Tek diluent (1W-1000; IHC World, Woodstock, MD, USA); nuclei were counterstained with 0.0012 µM DAPI (D3571; Invitrogen, Carlsbad, CA, USA). Stained samples were maintained and imaged in PBS. Image acquisition was done using a LSM 780 laser scanning confocal microscope (Carl Zeiss Microscopy, Jena, Germany). Three-dimensional reconstructions were carried out using Imaris v9.5.0 (Bitplane AG, Zürich, Switzerland).

### 2.16. Cinnamic Aldehyde Loaded Macrophage-RASMC Co-Culture Immunostaining and Microscopy

Synchronized RASMC (seeded at 5 × 103 cells in 96 well glass bottom imaging plate, 655891, Greiner Bio-One) were treated with CA, or RAW 264.7 macrophages pretreated with CA-ARAPas. Treatments were at a ratio of 1:30 RASMC to macrophages. After 24 h, cells were fixed in 2% paraformaldehyde, and permeabilized with 0.1% Triton-X (X100; Sigma-Aldrich, St. Louis, MO, USA). Cells were then probed for Nrf2 (1 µg/mL, ab137550; Abcam, Cambridge, UK) and alpha smooth muscle actin (1:200, 48938; Cell Signaling Technology, Danvers, MA, USA). This followed by AlexaFluor555 goat anti-rabbit IgG (4 µg/mL, A21429; Thermo-Fisher Scientific, Waltham, MA, USA) and AlexaFluor647 goat anti-mouse IgG (2 µg/mL, A21236; Thermo-Fisher Scientific, Waltham, MA, USA). All antibodies were diluted in IHC-Tek diluent (1W-1000; IHC World, Woodstock, MD, USA); nuclei were counterstained with 0.0012 µM DAPI (D3571; Invitrogen). Stained samples were maintained and imaged in PBS. Three-dimensional image acquisition was done using an LSM 780 laser scanning confocal microscope (Carl Zeiss Microscopy, Jena, Germany). Three-dimensional reconstructions were carried out using Imaris v9.5.0 (Bitplane AG, Zürich, Switzerland).

### 2.17. Statistical Analysis

Numerical data are represented as means ± SEM. Statistical analyses were performed using either a one-way or two-way ANOVA, followed by Tukey’s post hoc test, as appropriate with a *p*-value < 0.05 considered statistically significant (OriginLab, Northampton, MA, USA).

## 3. Results

### 3.1. Pluronic L101 Forms Micelles in Solution at Low Concentrations

**Figure 1:**
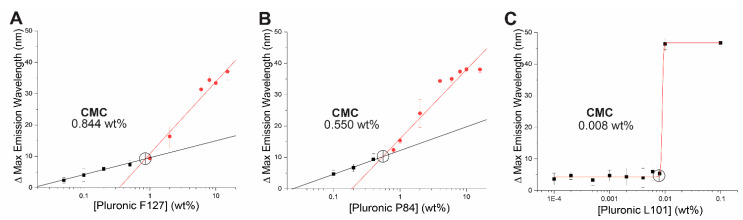
Varied PPO-PEO chain length directly affects critical micellar concentration of pluronic tri-block copolymer candidates. Critical micellar concentration was determined using nile red probe assay in phosphate buffered saline pH 7.4 for (**A**) Pluronic F127 (**B**) Pluronic P84 (**C**) Pluronic L101. Data represented as means ± SEM (*n* = 3 independent experiments).

Successful translation of nanotherapeutics to the clinic depends largely on overall toxicity of the formulation components. The tri-block copolymer, pluronic, has the potential to impact cell viability directly [26]. As such, it is critical to determine the concentration of polymer required to form micelles in solution, or critical micellar concentration (CMC). Generally, the lower the CMC, the lower the toxicity to cells. CMC of pluronic of varied PPO-PEO chain length differ [27]. In PBS at physiological pH, the CMC of Pluronic F127, Pluronic P84, and Pluronic L101 was determined as 0.844 wt% (Figure 1A), 0.550 w% (Figure 1B), and 0.008 wt% (Figure 1C), respectively. Pluronic L101 forms micelles in solution at remarkably low concentrations and hence has the highest likelihood of forming micelles with low toxicity to cells.

### 3.2. Synthesis and Characterization of Cinnamic Aldehyde Antioxidant Response Activating nanoParticles

**Figure 2:**
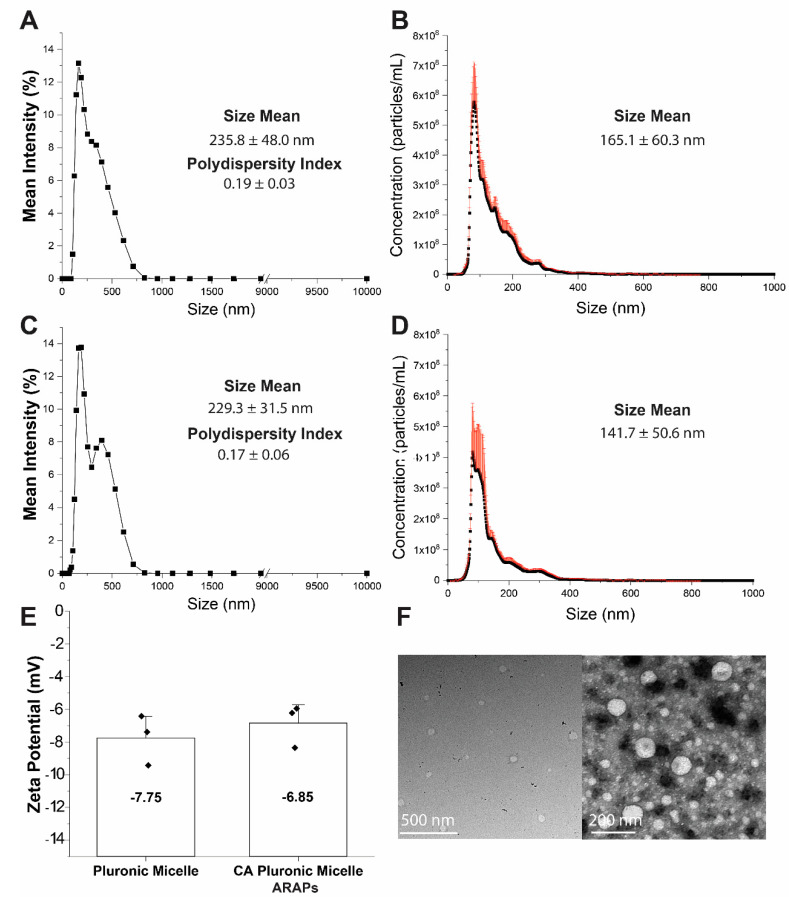
Nanoparticle characterization of drug free compared to cinnamic aldehyde encapsulated micelles (CA-ARAPas). (**A**) Dynamic light scattering plot of CA-ARAPas with reported size and polydispersity index. (**B**) Nanotracking analysis plot of CA-ARAPas with reported mean size. (**C**) Dynamic light scattering plot of drug free pluronic micelles (PM) with reported size and polydispersity index. (**D**) Nanotracking analysis plot of drug free PM with reported size. (**E**) Zeta potential values of drug free PM and drug loaded ARAPas. (**F**) TEM of CA-loaded ARAPas. (**A**,**C**) represented as mean (of mean intensity %) of *n* = 3 independent experiments. (**B**,**D**,**E**) represented as mean ± SEM (*n* = 3 independent experiments).

Nanoparticle characterization is an essential step in biomaterial research, which ensures reproducibility and control of batch to batch variation. Nanomaterials undergo characterization in various aspects, including size, size distribution, surface potential, and entrapment efficiency; all of which inform on the potential of the NP formed for cell loading. Dynamic light scattering (DLS) and Nanotracking analysis (NTA) are both techniques utilized to determine the hydrodynamic radius of the nanoparticles formed as well as the distribution of particle radii across populations, typically represented as polydispersity index or PdI. PdI values lower than 0.2 are typically considered consistent with sample homogeneity and highly desired for nanomaterial application. DLS reveals that the CA-ARAPas are slightly larger at 235.8 nm (Figure 2A) compared to drug-free micelle at 229.3 nm (Figure 2C). Similarly, NTA mean size for CA-ARAPas is slightly larger at 165.1 nm (Figure 2B) when compared to drug-free PM at 141.7 nm (Figure 2D). Both CA-ARAPas and drug-free PM have similar PdI at 0.19 (Figure 2A) and 0.17 (Figure 2C), respectively. Surface potential is another critical characteristic that impacts biological-material interaction. Surface potential of CA-ARAPas is slightly more positive than the control micelle (Figure 2E). Conventional TEM shows spherical nanoparticles between 100 and 200 nm (Figure 2F). Finally, using Equation (1), we calculated entrapment efficiency (EE) of CA in the PM to be 7.6%, much lower than the EE in PM of drugs of similar hydrophobicity [28].

### 3.3. Cinnamic Aldehyde Micelles Inhibit Vascular Smooth Muscle Cell Activation

**Figure 3:**
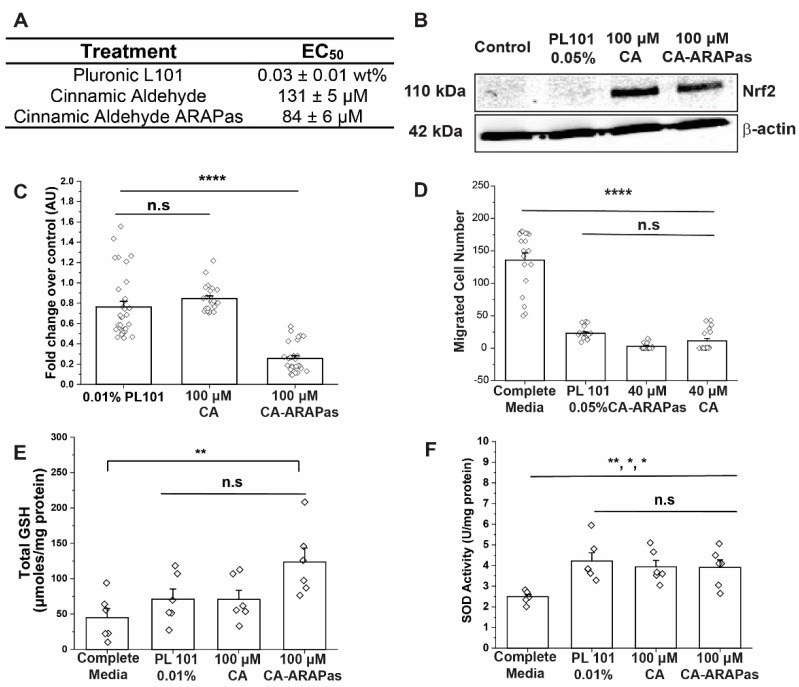
CA and CA-ARAPas decrease vascular smooth muscle cell proliferation, migration, with Nrf2 activation and production of antioxidants. (**A**) Summarized smooth muscle cell half-maximal response to treatment with CA, Pluronic L101 and CA-ARAPas (at 0.01 wt% of Pluronic L101). (**B**) Immunoblotting for Nrf2 in smooth muscle cells after treatment with CA, Pluronic L101 and CA-ARAPas. (**C**) Effect of treatment with CA, Pluronic L101 and CA-ARAPas on smooth muscle cell viability. (**D**) Quantification of cell migration in a standard scratch assay. Effect of treatment with CA, Pluronic L101 and CA-ARAPas on (**E**) Total intracellular glutathione (**F**) superoxide dismutase activity. Data represented as mean ± SEM (*n* ≥ 3 independent experiments in duplicates or triplicates) * *p* ≤ 0.05, ** *p* ≤ 0.01, **** *p* ≤ 0.0001, and n.s. not significant.

Cinnamic aldehyde has been previously reported to inhibit VSMC migration and proliferation in vitro [9]. As such, we aimed to determine whether CA-ARAPas exhibit a comparable or an enhanced effect in VSMC. To assess treatment effect on VSMC proliferation and viability, an MTT assay was used. Half maximal effective concentrations (EC50) were determined to be 0.0259 wt% for pluronic L101, 130.89 μM for CA, and 83.66 μM for CA-ARAPas (prepared at an L101 concentration of 0.01 wt%) (Figure 3A). Further, treatment of VSMC with 100 μM CA-loaded ARAPas shows a marked effect on proliferation compared to 100 μM CA, or pluronic L101 treatment alone (Figure 3C). The effect of treatment on migration of VSMC was assessed using a standard scratch assay. Treatment of VSMC with CA, Pluronic L101 and CA-ARAPas significantly reduces VSMC migration compared to untreated control (Figure 3D). Immunoblotting of treated cells reveals that CA and CA-ARAPas increase the level of Nrf2 protein (Figure 3B). Interestingly, PM alone does not consistently activate Nrf2 in VSMC (Figure 3B and Figure S2). Since Nrf2 activation is expected to increase the cell’s antioxidant defenses, we evaluated the effect of CA and CA-ARAPas on superoxide dismutase (SOD) activity and total glutathione levels. Treatment of VSMC with CA, Pluronic L101 and CA-ARAPas, all significantly increase SOD activity (Figure 3E), while only CA-ARAPas treatment significantly increases total glutathione (GSH) (Figure 3F).

### 3.4. Pluronic Micelles(PM) Are Feasible Particles for Macrophage Loading

**Figure 4:**
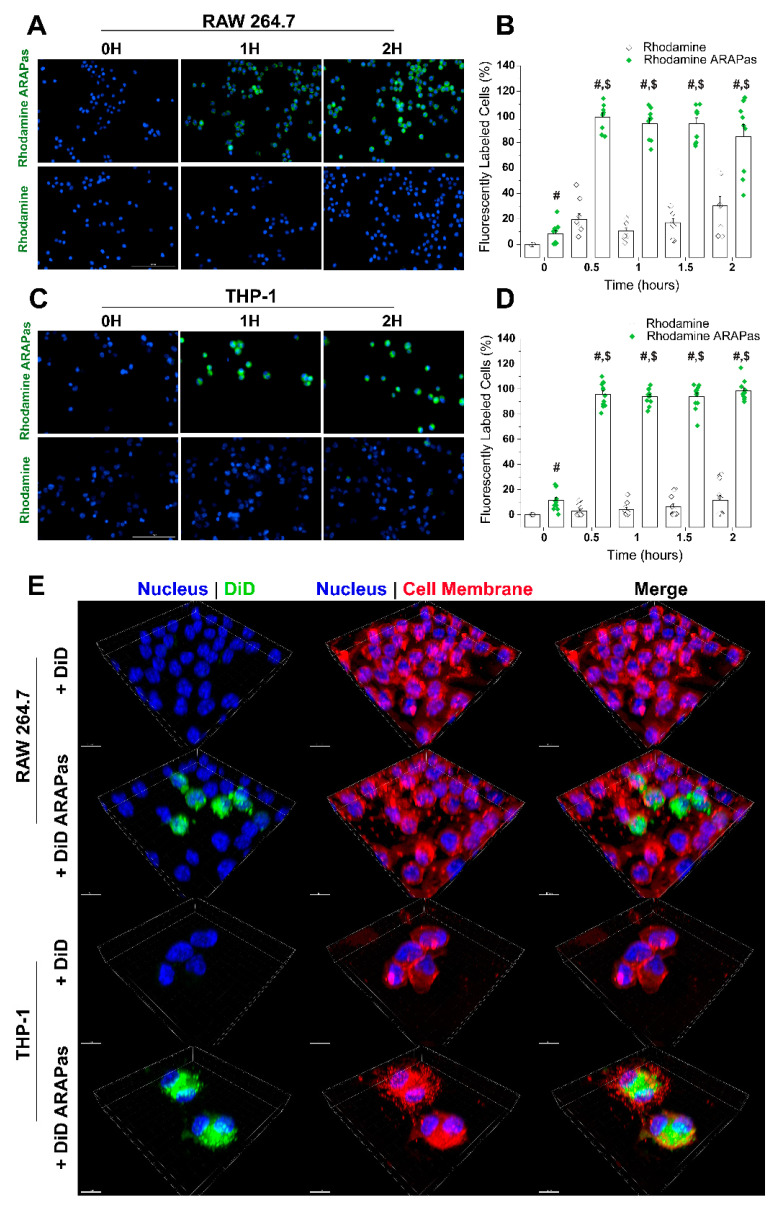
Pluronic micelles are internalized by macrophages in a time-dependent manner. (**A**) Representative images of a time course treatment of RAW 264.7 murine macrophages with rhodamine ARAPas or rhodamine alone. Scale bar = 100 µm (**B**) Quantification of fluorescent uptake in murine macrophages over two hours. (**C**) Representative images of a time course treatment of THP-1 human macrophages with rhodamine ARAPas or rhodamine alone. Scale bar = 100 µm (**D**) Quantification of fluorescent uptake in human macrophages over two hours. (**E**) Three-dimensional confocal microscopy was used to determine fluorescent ARAPas engulfment or internalization in RAW 264.7 murine and THP-1 human macrophages. Confocal images used are representatives of the results obtained in one independent trial. Scale bar = 10 µm. Data represented as mean ± SEM (*n* = 3 independent experiments in triplicates). # Significant with respect to rhodamine at the same time point; $ significant with respect to initial time point both with *p* ≤ 0.0001.

Macrophages are expert phagocytes that entrap a wide range of sizes of foreign material and bacteria [29]. Pluronic micelles have been known to accumulate in cells, depending on the PEO-PPO chain length and arrangement [30,31]. To use macrophages as drug delivery carriers, we must investigate whether macrophages can accumulate cargo-loaded micelles for transfer into target VSMC. With this in mind, rhodamine ARAPas were prepared and used to treat both murine and human macrophages for 0, 0.5, 1, 1.5, and 2 h. Macrophages were also treated with an equivalent concentration of rhodamine alone. All samples were imaged using a widefield fluorescent microscope for fluorescent probe signal (in green) and cell nuclei (in blue) (Figure 4A,C). Proprietary software (Gen5) was used to determine the percentage of cells positive for fluorescence over the time points used. Results show that treatment with rhodamine-labeled ARAPas greatly enhances accumulation in macrophages, an effect that persists over all time points. Moreover, rhodamine rapidly accumulates in all cells, and exhibits maximal effect within 30 min and plateaus over the rest of the treatment time (Figure 4B,D). Notably, a similar effect was shown in murine (Figure 4B) and human (Figure 4D) macrophages, suggesting that the process is species independent. Three-dimensional confocal acquisition reveals cellular internalization of DiD-ARAPas and not free DiD after treatment for 30 min in both cell types (Figure 4E).

**Figure 5:**
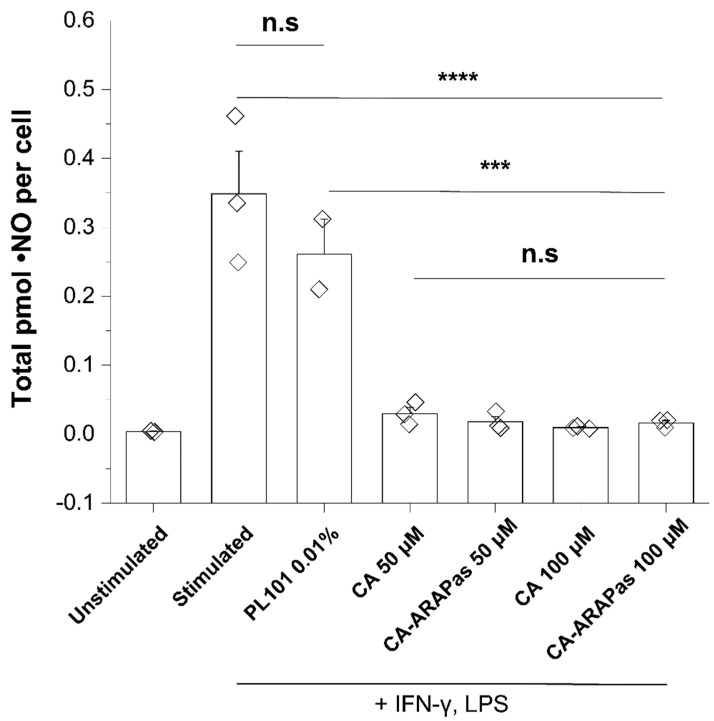
CA and CA-ARAPas abolish enhanced nitrite production in classically activated macrophages. Nitrite production comparison of treated stimulated and unstimulated macrophages. RAW 264.7 macrophages were either unstimulated or treated with 10 ng/mL IFN-γ for 7 h, followed by treatment with LPS (100 ng/mL) and PM, CA, or CA-ARAPas (50–100 µM) for a further 24 h. The cell media was analyzed for nitrite, and a total cell count performed. Data is represented as mean ± SEM (*n* = 3 independent experiments, *n* = 2 PL101 0.01% treatment) *** *p* ≤ 0.001, **** *p* ≤ 0.0001, and n.s. not significant.

Since macrophage recruitment to the sites of arterial injury contribute to inflammation during the arterial injury response, it was of interest to assess if the CA-ARAPa treatment modulates macrophage phenotype. Stimulation with IFN-γ and LPS results in classical activation of RAW 264.7 macrophages. One canonical measure of classically activated macrophages is overexpression of inducible nitric oxide synthase (iNOS), resulting in significantly greater production of nitric oxide (^●^NO), whose end-product is nitrite (NO_2_^-^). Previous studies by Kobayashi and colleagues [32] found that under classical activation conditions (IFN-γ/LPS), Nrf2 downregulated the expression of cytokines IL6, IL1β, IL1α, and the protein iNOS. We examined whether CA and its corresponding CA-ARAPas, as Nrf2 activators, would inhibit the enhanced nitrite production, an index of iNOS protein over-expression. We indeed found that CA and CA-ARAPas (50–100 μM) abolished the enhanced formation of nitrite associated with the classical activation-mediated increased iNOS protein expression (Figure 5).

### 3.5. Macrophages Effectively Deliver Cargo to VSMC in Culture

**Figure 6:**
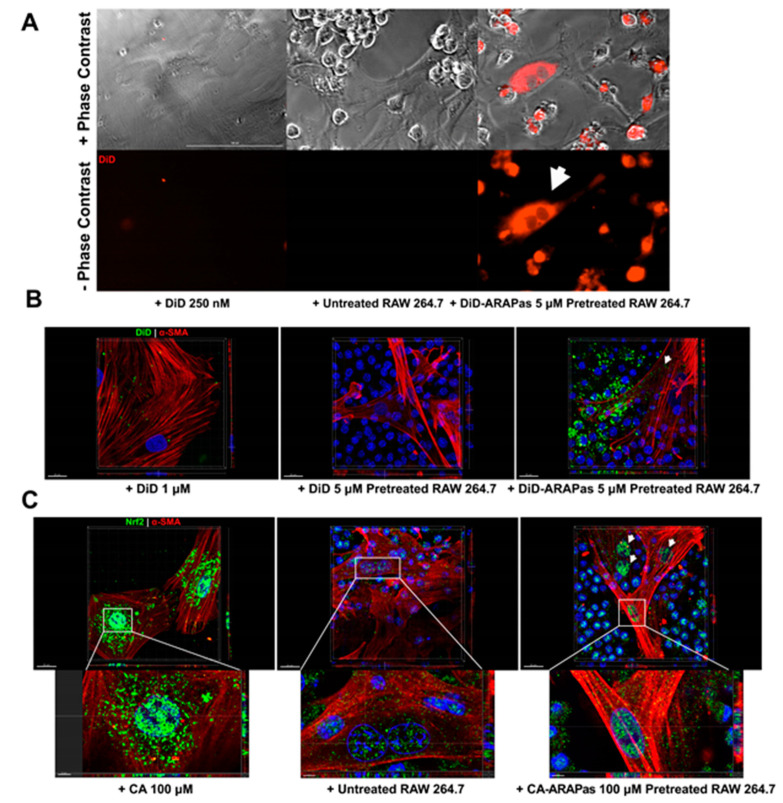
ARAPas treated macrophages effectively transfer cargo to vascular smooth muscle cells with Nrf2 translocation into the nucleus. (**A**) Outcome of kinetic co-culture of fluorescent cargo pluronic micelle loaded macrophages (1:10 ratio) with smooth muscle cells after 12 h. Scale bar = 100 µm (**B**) Three-dimensional confocal microscopy of DiD-ARAPas loaded macrophages with smooth muscle cells in co-culture after 24 h. Scale bar = 20 µm (**C**) Three-dimensional confocal microscopy rendering of Nrf2 immunofluorescent stained CA-ARAPas loaded macrophages with smooth muscle cells in co-culture after 24 h. Scale bar = 20 µm (top), 5 µm (bottom).

Macrophages are phagocytic cells quickly recruited to sites of inflammation, making them excellent systems to target VSMC in areas of vascular injury [33]. To investigate the potential of PM loaded macrophages for delivery of cargo to VSMC, these target cells were treated with macrophages loaded with DiD-ARAPas, and the kinetics of their interaction followed using live-cell microscopy. As shown, macrophage to VSMC transfer of fluorescence is visible after 12 h of treatment (Figure 6A). Additionally, three-dimensional rendering of VSMC and macrophage co-culture further supports macrophage to VSMC transfer of fluorescent cargo (in green) associated with alpha smooth muscle cell actin fibers (in red) (Figure 6B). Additionally, to determine the effect of CA-ARAPa-loaded macrophages in VSMC, these two cell types were co-cultured for 24 h. Immunofluorescence and three-dimensional confocal microscopy were employed to determine activation of Nrf2 based on subcellular localization after treatment. Results show increased Nrf2 nuclear signal in VSMC treated with CA-ARAPa-loaded macrophages, in stark contrast to VSMC treated with untreated macrophages at the same ratio (Figure 6C).

## 4. Discussion

We have successfully encapsulated a natural Nrf2 activator, cinnamic aldehyde, into micellar nanostructures formed by triblock copolymers. These nanostructures were termed Antioxidant Response Activating Particles (ARAPas) due to their ability to activate the Nrf2 pathway. CA-ARAPas effectively decrease in vitro features that contribute to neointimal hyperplasia, specifically VSMC proliferation and migration, while activating Nrf2 and increasing cell antioxidants. Additionally, CA encapsulation facilitates ex vivo macrophage uptake of the particles within minutes, contributing to a decrease in proinflammatory ^●^NO production. Importantly, feasibility for targeted macrophage-mediated delivery of CA to VSMC was shown based on macrophage to VSMC transfer of fluorescent cargo and Nrf2 nuclear localization after treatment in co-culture for 24 h.

The role of superoxide and other ROS as drivers of VSMC hyperproliferative and migratory behavior, directly contributing to neointimal hyperplasia and restenosis, has been well established [6,34]. Strategies that aim to directly modulate the redox environment at the site of intervention, such as in the case of Nrf2 activation, are desirable treatment options. CA is a naturally occurring class I Nrf2 activator, which we reported as an inhibitor of neointimal hyperplasia in a rat model when locally applied to the affected vessel [9]. However, in the clinic, access to vessels undergoing intervention can be restricted. Hence, treatment strategies that can be delivered systemically and target the area of interest are of high interest. For this purpose, we sought to explore encapsulation of CA into biocompatible nanoparticles. Pluronics (poloxamer) are commercially available triblock amphipathic copolymers that form micelle structures in solution. Of interest, pluronic micelles (PM) have been used to successfully encapsulate other small molecules with similar octanol-water partition coefficient to CA, e.g., paclitaxel [35,36], docetaxel [28]. Initially, three polymers were chosen based on their distinct hydrophile-lipophile balance (HLB) value and PPO-PEO profile. The chosen polymers F-127, P-84, and L-101 have a high, intermediate, and low HLB value, respectively [27]. Critical micellar concentrations of these polymers were determined using the fluorescent lipophilic probe, nile red. Interestingly, the CMC values obtained were quite dissimilar to those reported previously using probes like pyrene [27]. Ultimately, both pyrene and nile red-based CMC results agree that out of our polymer candidates, pluronic L101 has the lowest CMC, and its use for encapsulation of CA was studied further. CMC values are sensitive to environmental conditions such as pH, temperature, or ionic strength, and the properties of the fluorometric probe. Nile red has recently been shown to provide CMC results that are slightly inconsistent; this is partly attributed to solubility and aggregation issues of the probe [37].

To better understand the characteristics of CA-ARAPas, their particle size and distribution, shape, surface (zeta) potential, and entrapment efficiency (EE) were evaluated in this study. The diameter of the micelles (solvent-PM and CA-ARAPas) was determined using DLS, and NTA. Both methods show only a slight mean size increase in drug loaded vs. solvent only PM, with an acceptable polydispersity index (PdI ˂ 0.2) all in agreement with previous studies on docetaxel PM encapsulation [28]. Mean NP sizes for NTA are consistently smaller than those obtained using DLS, which can be attributed to the significant contribution of a few larger particles to the overall sample scattering [38]. Surface potential of the micelles is moderately negative. CA-ARAPas moderate size (about 200 nm) [21], and negative surface potential [39] are in line with properties desirable for an NP designed for subsequent loading into macrophages. Additionally, studies suggest that PM enter into cells via clathrin-mediated endocytosis [40], suggesting the potential for accumulation into non-phagocytic cells, like VSMC. Unfortunately, entrapment efficiency of CA inside ARAPas is less than 10%, much lower than what has been previously reported for paclitaxel [35,36] and docetaxel [28] (50–80%). Entrapment efficiency of CA in ARAPas is also in stark contrast to other methods of CA encapsulation using poly (DL-lactide-co-glycolide) (PLGA) [22], (92%). Importantly, encapsulation using pluronic L101 may be unstable in aqueous solution given the sparse hydrophilic corona, which may favor aggregation (10 wt% PEO content, HLB 1–7 highly hydrophobic). However, we did not see large particle sizes in the timeframe of our experiments. A strategy previously employed to overcome these limitations is the addition of another pluronic polymer with a much higher wt% of PEO chain and low CMC, e.g., F-127, 70 wt% PEO chain, HLB 18–23 highly hydrophilic [35,36], which directly influences thermodynamic stability of micelles by increasing intermolecular van der Waals forces between hydrophilic segments and hydrogen bonding interactions with water molecules in the aqueous medium [41].

CA, a Nrf2 activator, has demonstrated its effects as VSMC inhibitor of proliferation and migration in cell culture studies [9,13]. In this investigation, we aimed to determine whether CA-ARAPas carry the same known properties of CA. First, VSMC response to treatment with pluronic L101 was investigated. Treatment with polymer alone shows a concentration-dependent decrease in VSMC viability, with a remarkable effect even at low concentrations (70% viability at 0.005 wt%). Treatment with CA also shows a concentration-dependent response as previously published [9], with its half-maximal response around 130 µM. VSMC viability does not sharply decrease between 0.01 and 0.06 wt%, with 0.01 wt% chosen as the concentration of polymer to assemble the ARAPas. Interestingly, treatment with pluronic L101 and with 100 µM CA, both cause a similar decrease in viability. This effect appears to be potentiated in the treatment with 100 µM CA-ARAPas. In a scratch wound assay, both pluronic L101 and 40 µM CA significantly reduce migration of VSMC when compared to the activated control, effect that is recapitulated with 40 µM CA-ARAPas. Additionally, immunoblotting reveals that, as predicted, treatment with either CA or CA-ARAPas activates Nrf2 in VSMC. Though VSMC proliferation and migration effects were expected to be primarily through CA-mediated Nrf2 activation, we have found that our polymer of choice for encapsulation has a marked effect on both parameters by itself without consistent Nrf2 activation. The effect of CA, PL101, and CA-ARAPas on migration is close to 100%. Hence, a possible synergistic or additive effect between components remains to be explored. As previously reported, CA treatment of ZDF RASMC increases protein levels of GCLC and SOD-1 [9]; this increases intracellular GSH and SOD1 enzymatic activity. In this study, treatment of RASMC with CA and CA-ARAPas significantly increases SOD enzymatic activity; interestingly, so does PL101 treatment. Additionally, in contrast with previous results, this study reveals that only treatment with CA-ARAPas significantly increases total GSH levels in RASMC.

Immune cell-mediated delivery as a nanotherapeutic targeting strategy has been explored in the context of cancer [42,43], and neuroinflammation [20,21]. Macrophages’ natural ability to home to inflamed tissues makes them feasible carriers to deliver redox modulators to vessels undergoing surgical intervention [33]. The mechanism by which these immune cells take up NP has been under investigation and seems highly dependent on the design of the particles. Specific properties such as size, morphology, surface charge, and the effect of the protein corona, all affect macrophage intake of the particles. NP have been found inside macrophages within 10 min of treatment [44]. Our results indicate that ARAPas are promptly found inside macrophages, and reach peak internalization within 30 min. This effect holds in both human and murine macrophages. Previous studies have postulated that the effect of drug-NP-loaded macrophages occurs through various mechanisms, which include direct macrophage to target cell interaction [45]. Our in vitro VSMC macrophage co-culture system confirms that target cells, VSMC, slowly accumulate labeled macrophage membrane, which becomes apparent after approximately 12 h. Additionally, co-culture employing CA-ARAPa-loaded macrophages appears to induce translocation of Nrf2 to the nucleus of VSMC, suggesting Nrf2 activation. Nrf2 increase in the nuclei of VSMC is dependent upon CA-ARAPas pre-treatment of the macrophages, as macrophages alone do not recapitulate this effect.

Our study is not without limitations. Here, we find that both CA and CA-ARAPas inhibit VSMC proliferation and migration. However, it is not possible at this time to determine whether the effect is attributed in any capacity to the encapsulated CA in the micelles or diffusion of free CA, given the dynamic nature of micellar systems. However, VSMC Nrf2 translocation to the nucleus induced by CA-ARAPa-loaded macrophages suggests the transfer of encapsulated CA and not free CA diffusion, since any free CA should be washed away before adding the loaded macrophages to the VSMC. Surprisingly, VSMC treatment with pluronic L101 also appears to inhibit proliferation and migration in a Nrf2-independent manner, suggesting that the CA-ARAPas could have Nrf2-independent effects as well. Lastly, the effectiveness of targeted macrophage-mediated delivery of cinnamic aldehyde to VSMC in preventing or reducing neointimal hyperplasia after surgical intervention remain to be elucidated in an in vivo model.

## 5. Conclusions

In this study, we successfully encapsulated CA in a nanoformulation based on the micellization behavior of the pluronic polymer L101 in solution. CA, and CA-ARAPas exhibit similar effects in VSMC proliferation and migration, and both activate Nrf2. We demonstrated the prompt loading of fluorescent-ARAPas in murine and human macrophages, and its subsequent transfer to VSMC in culture. Nrf2 activation occurred in VSMC treated with CA-ARAPa-loaded macrophages in vitro. Our results suggest that CA can be encapsulated in polymeric micelles and loaded ex vivo into macrophages that can naturally target sites of vascular injury for targeted delivery of CA.

## Data Availability

All data are contained in the manuscript.

## Supplementary Materials

The following are available online at https://www.mdpi.com/article/10.3390/antiox10050709/s1, Figure S1: MTT assay for VSMC treated with PL101, CA, or CA-ARAPas, Figure S2: Whole membranes for the Nrf2 WB.
