# Supplementary material for: Delivery of Cinnamic Aldehyde Antioxidant Response Activating nanoParticles (ARAPas) for Vascular Applications
Source: Antioxidants (Basel). 2021 Apr 29;10(5):709. doi: 10.3390/antiox10050709 (PMC8145619; doi:10.3390/antiox10050709)
Supplement: Supplementary file 1 [file antioxidants-10-00709-s001.zip › antioxidants-1185491-supplementary.pdf]

Delivery of Cinnamic Aldehyde Antioxidant Response Acti-vating nanoParticles (ARAPas) for Vascular Applications

Ana E. Cartaya [1,2,3,†] , Halle Lutz [3,†] , Sophie Maiocchi [1,2], Morgan Nalesnik [4], and Edward S. M. Bahnson [1,2,3,4,5,*]

[1]Department of Surgery, Division of Vascular Surgery, [2]Center for Nanotechnology in Drug Delivery, [3]Department of Pharmacology, [4] Curriculum in Toxicology & Environmental Medicine, [5]Department of Cell Biology & Physiology, and McAllister Heart Institute. University of North Carolina at Chapel Hill, NC 27599

† AEC and HL contributed equally and share first authorship

[*]To whom correspondence should be addressed:

Edward Moreira Bahnson, PhD.

Center for Nanotechnology in Drug Delivery

125 Mason Farm Rd, Office 2102. University of North Carolina at Chapel Hill

Chapel Hill, NC 27599

Tel 919-843-0842

Email: edward_bahnson@med.unc.edu

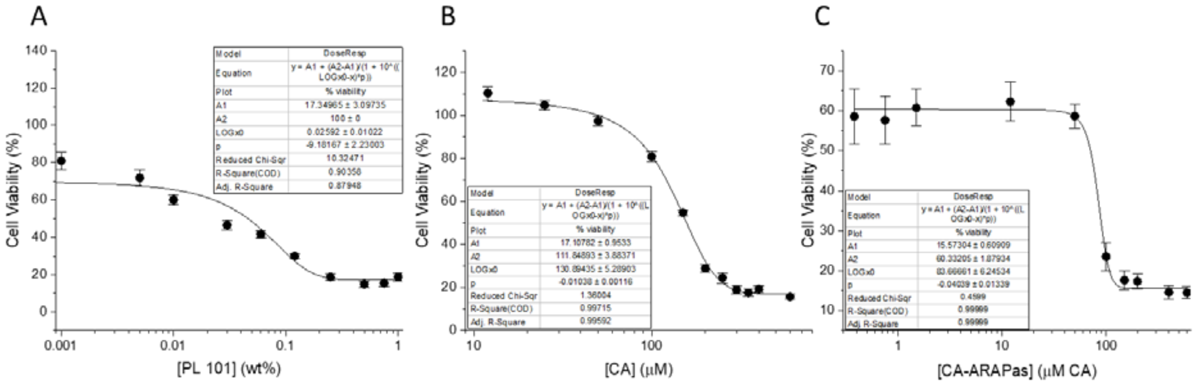

Figure S1. MTT assay for VSMC treated with PL101, CA, or CA-ARAPas. Vascular smooth muscle cell were treated with various concentrations of (A) Pluronic L101, (B) Cinnamic Aldehyde, and (C) CA-ARAPas (at 0.01wt% of Pluronic L101). Dose-Respose curve was fit using OriginPro and the EC50 calculated and presented in figure 3 of the main text.

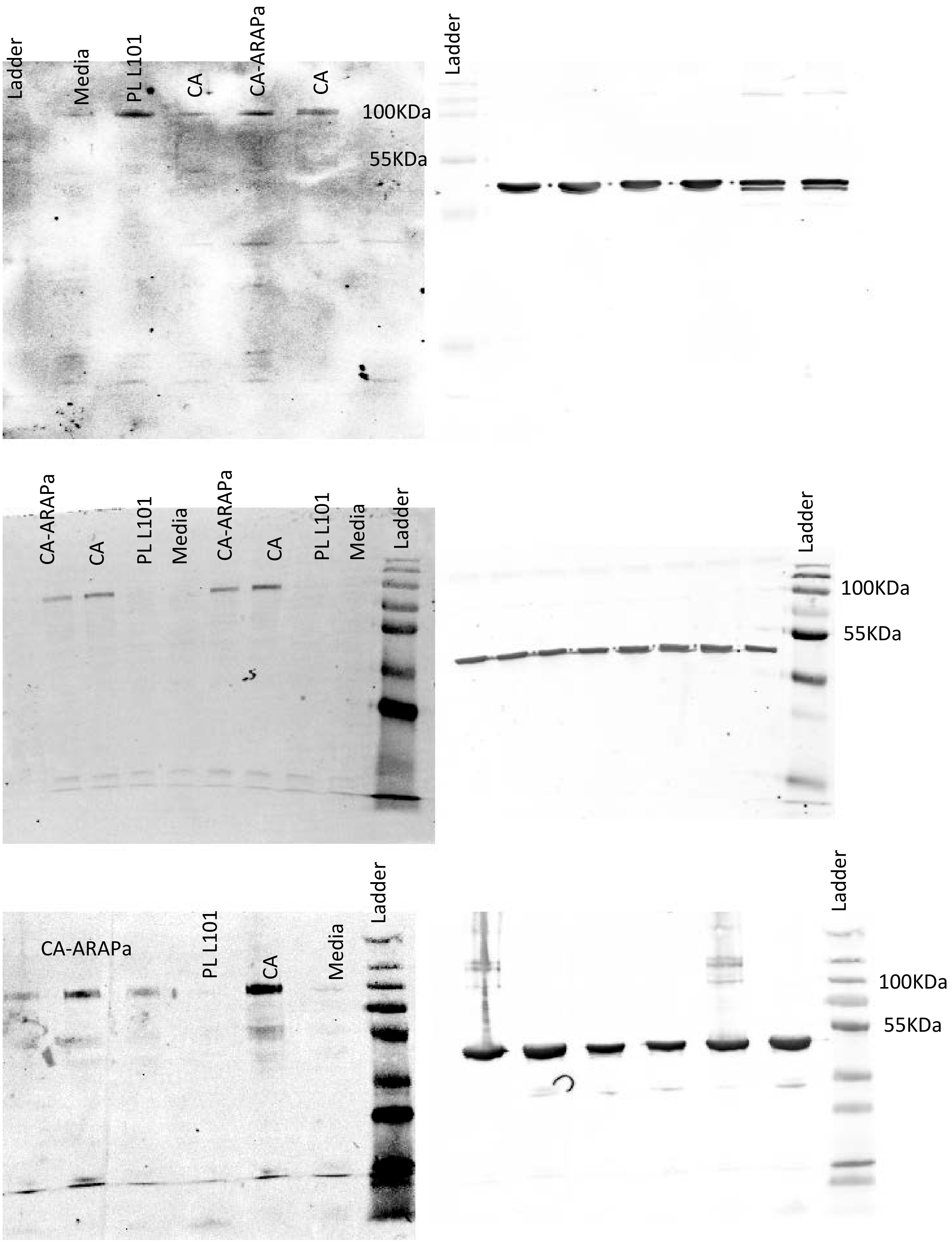

Figure S2. Whole membranes for the Nrf2 WB. Vascular smooth muscle cell were treated (A) Pluronic L101 (0.05%), Cinnamic Aldehyde (100 µM), or CA-ARAPas (Pluronic L101 0.05% - CA 100 µM). To the left, the mebranes were probed against Nrf2, to the right the same mebranes show the corresponding loading controls probing against β-actin.
